# Freedom and Anxiety: A Psychoanalytic Exploration of the Super-ego in the Postmodern Era

**DOI:** 10.1007/s43076-023-00303-w

**Published:** 2023-06-13

**Authors:** Sofia Jesus

**Affiliations:** 1 Psychology, Trinity College Dublin, Dublin, Ireland; 2 Loulé, Portugal

**Keywords:** Super-ego, Postmodern, Ego-ideal, Authority, Morality

## Abstract

The purpose of this study is to explore how the postmodern era may have altered the functioning of the Freudian super-ego. The aim of this paper is to create a discussion about which super-ego features may have disappeared, which may have been modified and which may have remained unaltered. Some of the hypotheses are illustrated through the discussion of the COVID-19 pandemic. The analysis suggests that postmodernity has modified aspects of the super-ego, promoting emancipation and the reduction of repression, while it generated uncertainty, new anxieties and an unconditional obligation to conform to unrealistic ideals of happiness and freedom.

## Introduction

The concept of super-ego emerged from the necessity to name and make sense of a set of shared experiences and feelings. Freud explored these experiences separately until he began to perceive them as being interrelated and circumscribed them to an all-encompassing concept. Thus, the abstract concept of super-ego comprises a set of different, autonomous and complex experiences: the experience of guilt; censorship; self-criticism; the experience of love, hate and fear between a child and his first object-choices; the idea of ego-ideal and of moral agency. Once these ideas were traced to a common denominator, the term super-ego was coined.

In *The Ego and the Id* (Freud, 1923), the super-ego appears as one of the three agencies of the psyche, alongside the id and the ego in his second topography. In this work, the author speaks of the super-ego and of the ego ideal (how the self wishes to be) as if they were interchangeable. The ego-ideal consists in the process of the child setting up an ideal in himself, to be sought after, through which he measures his actual ego. Another idea that Freud traced back to the super-ego concerns the moral agency of individuals. The moral compass is forged through the exposure to the do’s and don’ts in interaction with the parents, culture and educators. This prolonged exposure culminates in the introjection of the parental agency — this introjection constitutes the primordium of the super-ego.

From her extensive clinical experience, Klein (2002) proposed significant revisions to the classical theory of the super-ego. She identified pre-oedipal phantasies and anxieties regarding authority figures in children under the age of two, which led her to the conclusion that a primitive form of the super-ego was already present in the first year of life, contrary to what had been previously argued by Freud.

More recently on the subject of the super-ego, Lieberman (2019), an experienced clinical psychoanalyst, became motivated to write about the super-ego after having noticed that an increasing number of her patients’ issues were associated to a “new super-ego” phenomenon (p. 26) produced by contemporary culture. She remarks the new prevalence of symptoms of guilt, anxiety, extreme boredom, “psychic emptiness, powerlessness, and despair” (p. 26). Her contribution is discussed in-depth in the first chapter, alongside other recent contributions by authors such as Žižek, George Frankl and Lipovetsky.

The twenty-first century society is marked by the horrendous wars that happened in the twentieth century — an era characterised by an unprecedented number of deaths during conflicts. It also carries a new attitude towards existence on earth, due to an unparalleled technological advancement that has come to change the way people learn, spend their time, think and interact with each other. This society carries the spirit of change and revolution, and that is the reason why it challenges the super-ego, who is a carrier of tradition, fear, hate, love, guilt, aggression and obedience. The encounter of a traditional and ancestral entity such as the super-ego with a fresh society that discards all figures of the past is a phenomenon that will be examined in the following chapters of this paper.

In order to achieve this aim, this article is organised in two chapters. In the first chapter, the concept of postmodern super-ego is presented as well as the context in which it has emerged. This is followed by a description and analysis of the behaviour of postmodern Western individuals and the possible causes for the changes in their comportment and attitude. Then, the proposals made by contemporary authors in favour of a new super-ego are analysed. The following hypotheses are discussed: the break of the bond with the authority; the changes in the ego-ideal; the lack of a moral compass; the exemption from guilt; and the liberation of the impulses of the id.

In the second chapter, the coronavirus pandemic and compliance to government’s injuctions are used to illustrate the changes that may have occurred in the super-ego. The behaviour of the postmodern subject is analysed and divided in the following aspects: the break of the bond with the authority; the lack of super-ego censorship over the id and the uncertainty, anxiety and reflexivity that have afflicted postmodern Western societies. Finally, in the conclusion, there is a discussion concerning the renewed importance of the ego-ideal and the aspects of the super-ego that are considered to have been modified by postmodernity and those that were preserved.

## Chapter One: A Psychoanalytic Examination of the Postmodern Super-Ego

The concept of postmodern super-ego was coined by Slavoj Žižek (1999) to introduce the transformations that he observed in the moral behaviour of Western societies by the turn of the new millennium. Postmodernism emerged as a reaction against modernism and against the rational assumptions of the Enlightenment. According to Postmodernists, our rational and scientific values have only managed to lead mankind to wars and to an even greater state of poverty, prejudice and oppression. Postmodernism is characterised by a sceptical and nihilistic attitude towards universal notions, such as the notions of truth, reality and knowledge (Bauman, 1998). In brief, postmodernists have a tendency for self-referentiality, pluralism and irreverence.

In this social and cultural context, the Austrian-British psychoanalyst George Frankl, in *Foundations of Morality* (2001), identifies a series of mostly negative behaviours and attitudes that he uses to characterise the postmodern subject. The author identifies the increase of tendencies such as the emergence of limitless greed — defined as an “oral-cannibalistic drive” (p. 13) — the prevalence of selfishness and the liberation of sadistic-destructive impulses. Lieberman (2019) agrees with Frankl’s depiction of the postmodern society in her first chapter entitled “Analysing a ‘new superego’? Greed and envy in the recent age of affluence”. Similarly, Žižek (1999) describes the postmodern subject as being increasingly narcissistic, obsessed with freedom, self-realisation and enjoyment. While Frankl prefers to focus on the problem of the uncensored release of instinctual impulses, Žižek emphasises the postmodern cultural ideology that underlies these impulses. The latter perceives these changes as embedded in a continuous cultural evolution, while the former seems to consider that these changes are not only outside of culture but also constitute an attack on culture and on the established order. Albeit the two opinions are not contradictory. Both authors agree that there has been an overturn of traditional values and parental authority for the sake of freedom and liberation.

This dismissal of authority and tradition in favour of a personal and subjective code of conduct, susceptible to change from situation to situation, is characteristic of postmodern thought and has created a climate of moral uncertainty, in which every value, rule and moral code is constantly questioned and doubted. The reasons that motivated our behaviour in previous ages are now being disclaimed on the basis of a generalised nihilistic disbelief towards the values promulgated by the Enlightenment. However, as Frankl (2001) insists, we are not more certain of the reasons for our behaviour or about the values we should adopt than people of previous ages. Even though we have renounced the old ways, we have not created new purposes to replace them. The difficulty in creating new values in the twenty-first century comes from a widespread “state of helpless confusion” (Frankl, 2001, p. 03), disenchantment and cynicism in which people find it hard to believe that anything can be true or certain. Since tradition can no longer be relied on, everything becomes a matter of individual choice and mental life becomes dominated by an excess of possibilities. This reality portraits the contemporary phenomenon of “reflexivity” (Žižek, 1999), according to which every impulse is a matter of choice and everything is up for discussion.

The philosopher and sociologist Gilles Lipovetsky devoted his career to research the postmodern society. In his multiple works, he identifies in this society tendencies for exacerbated consumerism, hedonism, individualism and a loss of meaning of the major moral, social and political institutions. However, Lipovetsky does not reduce the current society to a complete deregulation of moral values and to a refusal of all past institutions like Frankl tends to do. On the contrary, he identifies a contradiction in the current society that neither Frankl nor Žižek explore and that is essential to comprehend the complexity and the state of confusion hovering over hyper-modern societies. In the *Crépuscule du devoir* (Lipovetsky, 1992), the author identifies in these societies a collective concern for morality, a concern for solidarity and righteousness, a struggle for ethical causes in a truthful demonstration of generosity. According to the author, this behaviour is in contradiction to the self-interested, ego-centred culture in question. Hyper-modern societies are often concerned about the future, about social minorities and human rights, and about climate change and other such causes. They are politically engaged and are capable of organising rallies that could last for days, in defence of a single individual who suffered an injustice at the hands of an authority figure. An example of this can be found in the protests led by the “Black Lives Matter” movement, against the death of George Floyd, on the 25th of May 2020. This depiction of reality does not necessarily contradict George Frankl and Žižek. In truth, it supports the idea that the postmodern subject is obsessed with freedom, justice, self-realisation and enjoyment, even though it apparently contradicts the features of selfishness and narcissism. However, it is possible that individuals will act in an extremely selfish and narcissistic manner if their freedom is being threatened. This seems to indicate that moral values persist in the current societies and that, in addition, they remain quite severe. However, they impose freedom instead of repression and containment. It is also possible that the imposition of freedom is still an imposition and may quickly turn into repression. Hence, on the one hand, we find a flexible, pragmatic and liberal dialogue about morality while, on the other hand, we find binary, rigorous and unrealistic claims that cause repression instead of realistically opposing it. An example of the latter is the concern with political correctness that reveals a “moral neo-conformity” (Lipovetsky, 1992) that adopts rigid strategies to dogmatically defend minorities.

Unlike Žižek, George Frankl (2001) offers multiple explanations and possible causes for this generalised transformation of behaviour. According to Frankl (2001), this new morality is a result of the colossal failure of traditional values during the twentieth century, which is exemplified by the numerous wars, increased poverty, unemployment, prejudice, oppression and extermination camps. Frankl (2001) argues that “(…) we cannot trust our judgements or even our perceptions because we cannot trust the concepts of our civilisation which have determined our judgements” (p. 07). These events of the twentieth century have brought upon a wave of disenchantment that has corrupted the bond between individuals and authority figures. The authority figures under consideration here are not just governors and law enforcers, but also God(s), parents, teachers and anyone who might be in a position of power over us. This relationship, instead of being marked by respect, fear and admiration, is now characterised by anger, scepticism, distrust and uncertainty. This patriarchal bond is at the foundation of the super-ego and that is why Frankl (2001) claims to be witnessing the “murder of the super-ego” (p. 29).

Frankl, Lieberman and Žižek postulate that the super-ego has undergone significant alterations, which are at the origin of the behaviour of the postmodern subject, and that these are not mere cultural changes. Some of these alterations influence not just the super-ego but the entire balance of the psyche. The most radical hypothesis was suggested by George Frankl, who advocated that the super-ego has been annihilated. Žižek, in a less radical stance, argues that there has been a disintegration of the old stability and a decline of parental authority which have significantly altered the super-ego.

### The Super-Ego in the Postmodern Era: A Static, Renewed or Annihilated Structure?

The following paragraphs present several proposals concerning the ways in which the super-ego may have been altered by the postmodern era. In order to do this, the concept of the super-ego is disassembled to reveal the multiple ideas it encloses.

It is George Frankl’s (2001) contention that, while traditionally aggression towards the authority and the super-ego was repressed and projected against the ego or against others, as postulated by Anna Freud (1936), now it is directed at the super-ego with the purpose of annihilating it. Frankl posits that the savagery displayed by the two world wars and by the horrors of Auschwitz has brought disappointment and apprehension towards the future. In addition to this disappointment, these events also elicited anger and aggression against the “father-figure which promised so much and failed so miserably” (Frankl, 2001, p. 7). According to Freud, one of the aims of the super-ego is precisely to repress the anger against the “father-figure” and help preserve the relationship with the love-object. If the anger is such that the super-ego is destroyed, there is nothing preventing the subject from acting-out his aggressive impulses. George Frankl applies this paradigm to the Middle Ages when people admired and glorified their king while, at the same time, they hated the power he had over them and envied his wealth and his privileges. The people’s love for their king was usually strengthened by killing the king’s enemies and by hating other kings. This displacement mechanism allowed citizens to discharge their aggression and not incur in the forbidden murder of the “own father”. According to contemporary Freudian theory, the current lack of displacement that Frankl points out, or the directness of the aggression, means that the pressure to express the anger has surpassed the benefits or the capacity to suppress it. In that scenario, the individual is thrown into the middle of an active and uninhibited Oedipus complex which could have catastrophic consequences. In this scenario, the annihilation of the super-ego is favoured, on the one hand, by the increase in aggression towards the father figure and, on the other hand, for the lack of inhibition of the Oedipus complex.

Nonetheless, the super-ego is not restricted to the functions of suppression of the Oedipus complex and regulation of the relationship with the authority. If that were true, the damaging of that relationship would be enough to annihilate the super-ego. On the contrary, the super-ego also includes a self-observing and self-criticising agency — the ego-ideal — which, even though it is formed in the relationship with the other, it mostly manages the relationship of the individual with himself. That agency alone can generate a great deal of shame and anxiety. Furthermore, if the super-ego was no longer a structure in the psyche or if it had been modified, then likely we would note the absence or the modification of the ego ideal, as well as of the feelings it produces. An indication of the state of the ego ideal could be then provided by the preponderance of shame in society. On this subject, while Frankl would probably be of the opinion that shame is experienced less often and with minor intensity; Žižek is likely to argue, in view of the increase of opportunities for social comparison on social media, that shame is on the rise. Consequently, it is possible that the ego ideal is becoming stronger while the super-ego is weakening, causing individuals to feel shame rather than guilt. In addition, considering the fact that the ego-ideal has recurrently been associated with narcissism in the past (Freud, 1991; Rosenfeld, 1952) and that the postmodern society has been described as predominantly narcissistic, it could suggest that the ego-ideal has taken command of the psyche in detriment of the super-ego. This hypothesis could explain how anxieties can persist even though the bond with authority has been damaged.

The third hypothesis being analysed also represents a structural change of the psyche. This hypothesis consists in the idea that the id has been liberated from the oppression and censorship of the super-ego. Since censoring the id is pointed out as one of the main functions of the super-ego (Freud, 1900), its absence can constitute an indicator of the disappearance or weakening of the latter. According to Frankl, this liberation is attested by the unconstrained behaviours of postmodern societies. The author entitled this phenomenon “ego mania”: a state where the id runs free. He devotes an entire chapter to the description of “the breakthrough of the repressed”, in which primitive areas of the psyche, such as aggressive, narcissistic and sexual drives, previously repressed and sublimated, now find unrestrained expression. Frankl is not the only author claiming that there has been an increase in behavioural permissiveness and an ease of obligations and restrictions after the Second World War. This idea is supported by both Lieberman and Lipovetsky in several of their works. Žižek does not strictly disagree with Frankl’s claim, he agrees that a new array of behaviours has become available and that individuals are no longer culturally required to meet traditional expectations of society. Nonetheless, he claims that individuals often still feel an inner pressure to please by conforming to those same expectations. The argument of the liberation of the id does not necessarily support the idea of the annihilation of the super-ego, contrary to what Frankl advocates. The main reason why this conclusion does not follow from this argument is that the instinctual drives of the id are not being expressed without constraints; on the contrary, the freedom they impose generates a great deal of anxiety whenever individuals attempt to balance their obligations versus their freedom to gratify their desires.

While evaluating the state of the super-ego, Frankl postulates that in our present time there is an absence of a moral compass which relates to an alteration of the super-ego. These notions are acquired through the identification to and introjection of the do’s and don’ts of the parental agency. Even though Frankl argues that individuals in the twenty-first century lack a moral compass, he did not explain how nor when this change came to be. If we accept the emphasis postmodernism has placed on individualism and on the obligation to be free, as well as how it has overturned all moral institutions, then it is understandable that individuals feel a lack of a moral compass. The freedom to make one’s own choices only increases the uncertainty and difficulty of making them, especially when compared with the simplicity of following tradition or a religious text that pre-establishes the aim of life and the way to attain that aim.

#### The Issue of Guilt

The lack of a moral compass brings us to another indicator of the state of the super-ego: the preponderance of the feeling of guilt. The super-ego produces guilt to force the ego and the id into conforming to certain idealised standards. Therefore, if the super-ego has been annihilated that should cause the feeling of guilt to disappear. The experience of remorse appears with the depressive position in response to the damaging attacks or phantasies about such attacks done to the parental figure. The intensity of the guilt is a good indicator of how much the super-ego is overpowering and, often, an indicator of the amount of aggression that is being repressed. When guilt is experienced, the feeling of love and admiration surpasses the anger and resentment, and the individual begins to wish to make reparations for the previous sadistic attacks. However, Frankl postulates that if the bond of love with authority has been damaged, then remorse is not experienced, and anger is free to express itself. According to Frankl, this scenario illustrates the current state of Western civilisation: he argues that individuals no longer experience guilt or experience it far less than before the twentieth century. On this important topic, Žižek, Lieberman and Frankl are not in agreement. Žižek and Lieberman consider that the postmodern super-ego has generated new and increased forms of anxiety.

Even though the link between super-ego and guilt is strong and undeniable, there is another important aspect to consider. The absence of guilt does not necessarily mean the super-ego has been annihilated. If we consider the double-faced super-ego conceived by Klein, an absence of guilt could mean that we are faced with a primitive and sadistic super-ego, which has not yet attained the depressive position. Indeed, Frankl’s description of the behaviours currently happening under what he considers to be an absent super-ego seem to correspond to the behaviours found under a pathological super-ego as described by Bion (1967), or to the primitive Kleinian super-ego. Assuming the super-ego is created when the first bad object is introjected, would it still be considered a super-ego if the sadistic phase never ended and the individual was forever without remorse? If the super-ego never developed past the introjection of bad objects, would that entity, constituted by a bad thing in itself that instigates fear and retaliation, still be considered the primordium of the super-ego? If the super-ego can exist without the experience of guilt, then the argument presented by Frankl cannot be validated.

In any case, this discussion is only relevant if we consider that societies are now guilt-free, which Žižek and Lieberman do not. According to Žižek, the postmodern super-ego has brought upon a peculiar and unique obligation — the obligation to be free and to enjoy life. This order, which resembles a direct commandment from the id, generates “new guilts and anxieties, instead of opening up a brave new world in which we can enjoy shifting and reshaping our multiple identities” (Žižek, 1999).

The previously mentioned liberation of instincts brought by postmodernity is only apparently free of restrictions. In Žižek’s publication in the London Review of Books entitled “You May!” (1999), the author argues that there are no “strong prohibitions in a society awash with permissiveness”. However, there is also no escaping from the excessive freedom it conveys. Thus, “You May!” becomes mandatory, it becomes “you must”. There is a call for an unconstrained transgression with the promise of suspension of judgement. Yet, if individuals are being encouraged to have the maximum pleasure by enjoying themselves to the fullest, then they must enjoy, otherwise, if they feel that they could have enjoyed more or feel unable to enjoy, it will result in a feeling of guilt for failing to be happy. If culture no longer imposes obligations, postmodern individuals now impose them to themselves and feel compelled to enjoy them, since they are the ones “choosing” to fulfil them. The postmodern society is falsely permissive since it is replete with regulations and impositions under the false pretence of ensuring our happiness.

These new anxieties mentioned by Žižek resemble a recently conceptualised social anxiety, known popularly and in the field of psychology as *FOMO* — fear of missing out, which is similar to *FOBO* — fear of better options. FOMO is described as the anxiety of being disconnected, of being excluded, of missing an opportunity for social interaction, of not making the right choices in a paradigm where every experience must be enjoyed to the fullest. FOBO is characterised by a feeling of anxiety, frustration, stress and unhappiness. These result from the excess of freedom of choice, which becomes problematic for the postmodern subject because he has no fixed criteria to help him decide. These two terms, coined by Patrick McGuinnis, in 2004, are indissociable from an unhealthy use of the internet and social media.

Lieberman (2019) agrees with Žižek on the subject of guilt. From her recent clinical observations, she concludes that her patients exhibit more feelings of guilt than ever before. To illustrate this reality the author gives the example of practices of self-starvation, excessive exercise and unreasonable diets which serve to punish the ego, control the impulses of the id and force the individual to conform to an unrealistic ideal. All these practices follow the often-heard expression “no pain, no gain”.

Previously, it was shown how we could conceive the absence of guilt without that implying the absence of the super-ego. However, another scenario should be considered in which the super-ego has been annihilated, as Frankl argues, but the sense of guilt persists, as Žižek contends. If Žižek’s contention holds true and the postmodern society is suffering from additional anxieties, how can that idea be reconciled with the theory of the annihilation of the super-ego? Shouldn’t the disappearance of the super-ego suppress these anxieties? In other words, is it possible to experience guilt without a super-ego? The formulations made by Freud, Klein, Bion, Winnicott and many others are not favourable to this hypothesis since the super-ego is pinpointed as the main generator of guilt.

However, in one of his writings, Žižek (1999) has managed to explain how we can conceive the perseverance of the feeling of guilt in the absence of the super-ego. In the postmodern era, with the purpose of self-assertiveness and self-fulfilment, there is, according to Žižek, a tendency for a positive rewriting of the narrative of our past, as a way to surpass traumatic or unpleasant experiences. The same solution is applied to the traditional rules of society whenever they are considered too severe.

Žižek illustrates this idea with a sarcastic and rhetorical question that ridicules the liberalism of postmodern thinkers: “Is one of the Commandments too severe? Well then, let’s regress to Mount Sinai and re write it: adultery – fine, provided it is sincere and serves the goal of profound self-realisation” (Žižek, 1999). There is a sort of collective illusion of omnipotent control over life, rules and personal emotions. It is an illusion because the rewriting of a moral rule or emotional experience does not really erase nor alter the consequences the real experience had in the first place. In other words, “what disappears is not ‘hard fact’ but the Real of a traumatic encounter whose organising role in the subject’s psychic economy resists its symbolic rewriting” (Žižek, 1999).

This proposition is very important for the understanding of how culture can change without it necessarily altering the functioning of the psyche. For example, if we consider that postmodern ideology has changed the way the new generation cares for the elderly family members, which is an idea Žižek entertains, particularly towards a disengagement and a lack of accountability, then, according to Žižek, that would not absolve our moral conscience of the feeling of guilt. Therefore, if the super-ego has faded from our psyches, there could be a time lag between its fading and the disappearance of its effect, i.e. the feeling of guilt. In addition, the fact that the learning of the do’s and don’ts depends less on culture and more on the transmission of the unconscious processes of the parent’s super-egos (Laplanche & Pontalis, 1988) reinforces Žižek’s proposition. In sum, the feeling of guilt may persist even after the rules that cause that feeling have been overturned and even after the psychic entity that produces that feeling has lost its ground.

### Discussion

Some of Frankl’s arguments have found more support than others in the psychoanalytic community. Authors agree that the overturn of authority figures implies that there has been a liberation of the id from super-ego oppression. However, not all authors agree that there has been an unconditional liberation of impulsive drives, since, as Žižek observed, the postmodern subject is not entirely free from restrictions, obligations and traditional expectations. In brief, the id may have been liberated from super-ego oppression, since it is not as often repressed. However, the expression of the recently liberated instincts is not exempt from guilt and shame. Additionally, the idea according to which there is a generalised state of helpless confusion, causing individuals to doubt their concepts of truth and falsity, right and wrong, is fully corroborated by several authors. All of the authors mentioned above are also in agreement concerning the idea that there has been an overturn of paternal authority, i.e. a break of the bond of obedience, fear and love with authoritarian figures, as well as a disbelief in moral and social establishments.

Nevertheless, as already stated, Frankl’s theory concerning a guilt-free society found a great deal of opposition. Finally, the idea that the super-ego has suffered changes or that it might be getting weaker is quite different from the idea of an annihilation of the super-ego, which is far more extreme and, consequently, more difficult to support. In the next chapter, these ideas are applied to a real and ongoing social problem, which took place worldwide. The illustration of the postmodern super-ego simplifies its analysis and puts to a test the aspects said to have been modified and the ones said to have remained unaltered.

## Chapter Two: An Illustration of the Postmodern Super-Ego Through the Discussion of the COVID-19 Pandemic

The aim of this chapter is to put to a test the hypotheses presented in the first chapter concerning the postmodern super-ego. The functioning of this agency can become apparent in the way individuals experience guilt; in the pressure to conform to an ideal; in the compliance to a code of conduct; and in the repression that is applied over instinctual drives. In this chapter, the challenge is to find a real and current scenario where these feelings and behaviours may surface and can be explored.

The year of 2020 was a very eventful year, from the killing of George Floyd, to the election of Joe Biden and the outbreak of a global pandemic. From these events, the COVID-19 pandemic constitutes the focus of this chapter for its global spread and for the powerful and unpredictable social responses it prompted.

When China reported its first death from the disease on January 11, very few people paid attention to this pneumonia-like virus. By year’s end, around 1.7 million people had died from this virus (World Health Organization, 2020). Countries’ responses to the spread of the disease varied, some were quick to implement lockdowns, social distancing procedures and close the borders, and others suspended these decisions despite pressure to adopt them.

When the first COVID cases started to appear in the Western part of the world, a feeling of generalised panic broke out. There was no knowledge regarding the modes of transmission, the level of infectiousness, the most common symptoms and, particularly, the mortality risk. Anything was possible, much like a dream or a science fiction novel, reality seemed distorted and dangerous, evoking Hannah Arendt’s (1958, pp. 2–3) accurate interpretation of the relationship between dreams and reality: “Science has realized and affirmed what men anticipated in dreams that were neither wild nor idle”.

This chapter is not intended to examine the full complexity of the events brought upon by this pandemic, on the contrary, much of this extremely complex phenomenon remains to be examined since it is being considered exclusively in so far as it illustrates the ideas posited in the previous chapter. In other words, this chapter focuses exclusively on what can be seen within the pandemic regarding behaviours that implicate changes in the super-ego.

### Break of the Bond with the Authority

More than 60 years have passed since Milgram’s world-shattering experiments on obedience have revolutionised the way humanity thinks about human behaviour. Many doubts remain, particularly, regarding the replication of these results in our present time. Some would argue that results would be different today, in other words, that more people would disobey authority after having been made aware of the dangers of blind obedience post world-wars. Both Freud (1918) and Georg Frankl (2001) point out the disappointment and distrust towards authority figures that followed the horrendous war period, which should have led to a break of the bond that is at the very core of the Freudian super-ego. When Burger (2009) partially replicated Milgram’s studies on obedience in 2009, he found that compliance rates in the replication were only slightly lower than those found by Milgram (1974). One could argue a lot has changed since 2009; however, is it possible to claim that individuals have become more disobedient or more resistant to authority? What is the current state of our obedience to authority? Is humanity becoming progressively more disobedient, cynical and free? Has the postmodern western individual fashioned his own autonomous and orphaned super ego? Are individuals really governed by Lipovetsky’s “principle of sovereignty”?

Another question left unanswered by Milgram’s experiments concerns how groups obey authority. The relationship to authority is a social process, therefore, an analysis of the acts of obedience and disobedience should be integrated in a societal level approach (Morselli & Passini, 2011). This societal level was neglected by social psychologists who focused their studies rather on destructive obedience (Fattori et al., 2015). More recently, the focus has shifted into studying specific acts of disobedience within a political framework, such as the Hong Kong protests, or the George Floyd protests. Nonetheless, studies of collective obedience are scarce. Does postmodern western society still endure gruesome impositions under the premise of obeying an authority?

In this scenario, the COVID-19 pandemic could be perceived as a unique test of obedience to authority, not just at a community level, but at a global level. During the pandemic, health authorities emitted guidelines and orientations that were converted into laws and mandatory guides of conduct by governments all around the world. Individuals were compelled to stay-at-home during long “lockdown” periods, if they were found in the streets without a permit or a legitimate reason they faced a fine; events and public gatherings were not allowed; face masks had to be worn in all public places and transports (even pregnant women were forced to give birth using face masks); hands and surfaces had to be sanitised regularly; a 2-m distance had to be kept from other people at all times; schools and workplaces were shut down; most external borders were closed, making it impossible to travel for non-residents; during special holidays, like Christmas, New Year’s and Easter, authorities forbade circulation within the country, so most people could not visit their relatives or friends. This list of restrictions goes on and differs according to each country’s specific regulations.

There can be no doubt that these restrictions were highly challenging and strained people’s mental health, causing social isolation, disrupted work and family routines, layovers, economic instability, promoting social anxiety, germaphobia, sedentarism and so on. In addition to being an encumbrance and having had multiple consequences, some of the directives emitted by some European governments revealed inconsistencies and contradictions, which could have planted the seed of distrust and led to revolt and noncompliance. For example, in France, in the beginning of the pandemic, the minister for health, Agnès Buzyn, declared facial masks to be useless for the general population, she explicitly said that the blue masks did not offer any protection (Bellier, 2020). She has also assured the population a full stock of chirurgical masks that would be distributed if they ever became necessary. On March 17th 2020, Sibeth Ndiaye reinforced the idea that the French population could not buy facial masks at the pharmacies because these were not necessary if one is not ill (Le Parisien, 2020). However, 2 months later, not only masks became mandatory in every public and enclosed space in France, regardless of one’s health condition, there was a shortage of facial masks, leaving the population and health professionals completely unprotected.

The pandemic situation and the degree of compliance to lockdown rules in the Western world differed greatly from one country to another, this is due to a series of factors that will not be examined in this paper. Even though some countries presented a high level of compliance, others have greatly disobeyed at least some of the measures implemented to stop the spread of the SARS-CoV-2. In addition, opinions regarding how the pandemic was handled and the necessity for the measures implemented varied greatly, dividing society in two antagonistic blocks. Thus, making it impossible to analyse it as a whole and arrive at a solid conclusion on our current state of obedience to authority. However, it is possible to analyse both scenarios — the scenario of compliance and the scenario of non-compliance — in regard to what they may suggest in what concerns the state of the postmodern super-ego.

In France, compliance rates to lockdown reflect distrust in governmental agencies (Sarracino et al., 2022), 60% of the questioned subjects have breached lockdown at least once. The French mainly used travel certificates for reasons other than those indicated (24%), walked beyond the authorised 1-h limit (17%), and saw family members in each other’s homes (23%) or friends (20%) (Milard & Mariot, 2021). In France, it was possible to observe the full strength of the postmodern super-ego. On the other hand, in the UK, the majority of the population were predicted to be full compliers (52.5%), 36.7% were frequent compliers and a small minority were occasional compliers (6.3%) (Wright et al., 2021). In Portugal, the majority of the participants (94.4%) obeyed the confinement (Almeida, 2020).

#### The Scenario of Non-compliance and the Full Strength of the Postmodern Super-Ego

There were large groups of people protesting COVID-19 rules in several countries, for whom the imposition of these rules was perceived as a form of authoritarianism, rekindling a pre-existing disappointment, anger and distrust towards authority figures. These feelings, which were described by Frankl (2001) as illustrative of the annihilation of the super-ego, do not just concern the bond with powerful figures but, instead, are embedded in the tissue of postmodern society and are often directed towards formerly reliable social institutions, such as the media, pharmaceutical companies and many other institutions that are now more often considered manipulative and unworthy of public trust. It may be possible that the aversion experienced by the non-compliers was not directed at the safety measures per se, in that they were merely trying to prevent the spread of the virus and ensure the protection of civilians, but were rather a reaction to the general authoritarian climate and modes of persuasion. Thus, anger would have been directed at authority as a construct and not at pandemic rules. This anger is reinforced by a feeling of helplessness and vulnerability which is a consequence of the murder of the own father and only protector.

At the same time, it appears that the struggle against authority has given individuals an enormous sense of power, representing the end of the Freudian childhood helplessness and, consequently, the independence from the need of a super-ego. The postmodern super-ego is the attempt to annihilate the super-ego. The current generalised disbelief in God only adds to this mass sentiment. The chosen protectors of society have become a threat by abusing their power — a power that, ultimately, we have attributed to them, making us somehow to blame and unworthy of trust as well. As a result, the disappointment felt towards the abusive father extends to a self-disappointment and a distrust in the justice and social systems that have created this (dis)order.

The disobedience demonstrated by these protesters is even higher considering that in the context of this pandemic it implied exposing oneself to being labelled as a conspirer, as a selfish person, it implied actually risking contamination by the SARS-CoV-2 virus and, depending on the country, risking a fine or criminal charges if caught disobeying. As Erich Fromm (1984, p. 21) puts it: “in order to disobey, one must have the courage to be alone, to err and to sin”. In some countries, like France, this protest movement was more prominent and taken seriously than in other countries, where it was quickly dismissed and labelled as a movement led by conspirators, pandemic deniers with pseudoscientific beliefs and health practices.

#### The Scenario of Compliance and the Regressive Super-Ego

In countries like Portugal, the percentage of compliance suggests that the postmodern super-ego may have regressed to a traditional and Freudian super-ego, better equipped to handle emergency situations, for such was the death menace of this pandemic. As long as individuals obey the rules, they are safe and protected. The mere suggestion of a threat of illness and death is enough to revive an almost religious fervour and servitude to the one offering shelter from harm, mending the broken bond with authority and forgiving all past faults.

In 2021, the Portuguese believed that more than 2/3 of the politicians were corrupt and that opportunities for corruption increased in the context of the pandemic (Sousa & Magalhães, 2021). Despite these considerations, the Portuguese compliance rate to government’s pandemic-related directives was tremendous, suggesting that the fight for democracy and anti-system stances are reserved for times of peace and stability. In troubled times, under significant pressure, the postmodern super-ego’s tendency towards liberation, freedom and enjoyment may become severely repressed, as Freud (1920) accurately predicted when he posited that all drives are conservative, in other words, they tend to regress to a previous and familiar state.

In countries where compliance reigned, the authority exercised by heads of government was seen as what Fromm describes as “rational authority” (Fromm, 1984, p. 20), which is implemented in the name of reason, and therefore is indisputable. Irrational authority on the other hand acts through force and suggestion. Of the two, the first is the most dangerous, since individuals are not forced to obey, the individual is under the illusion that he is acting voluntarily, that he follows only what is reasonable. And, as Fromm (1984, p. 47) wisely asks: “Who can disobey the “reasonable”? Who can disobey when he is not even aware of obeying?”.

Must we conclude that the postmodern super-ego is a fragile structure that may regress to previous states whenever needed or threatened? Not necessarily. There is another scenario where compliance to COVID rules upholds the idea of a postmodern super-ego, instead of indicating a regression. As Lipovetsky observed, hyper-modern societies have developed a concern for solidarity and righteousness; therefore, if COVID rules were understood as one’s obligation towards society, one’s obligation to do good and act morally, compliers could have been upholding their individual sovereignty by appropriating governments’ directives as their personal convictions. This can be understood as a way of obeying authority without recognising it, without compromising their individualism. Of course individuals could have been appropriating rules as personal convictions before the postmodern era; however, since now there is more freedom to manifest one’s opinion, to rebel, to disobey, individuals are compelled to make use of the freedom being given to them, augmenting the pressure to act based on personal convictions instead of merely following rules, which is something frowned upon.

### The Lack of Super-Ego Censorship over the Id During COVID-19

In the previous chapter, the function of censoring the impulses of the id, attributed to the super-ego, was said to have been suppressed or at least modified in the postmodern era**.** The liberation of the instincts of the id is particularly visible in the liberation of the aggression towards the paternal authority. Lieberman (2019) commented on how parents have been losing their authority over their children and, conversely, how children have been imposing their will on their parents. If the super-ego first appeared to suppress the Oedipus Conflict and to preserve the relationship with the menacing father, it seems to no longer be accomplishing that task.

During the COVID-19 protests in France, the relationship towards the father-figure (which is here being equated with the government) stops being “ambiguous” and becomes charged with aggression and disappointment. This occurrence is motivated, in part, by the events of the twentieth century, as Frankl claimed (2001) and encouraged by postmodern culture and ideology. The ideals defended by postmodern individuals are intrinsically conflicting with authorities and hierarchies which establish social inequalities and lead to injustices and abuses of power. In the current postmodern context, the values of subjectivity, individualism and liberation are now social imperatives that must be defended at all costs. Since authority figures are the ones who can hinder those aims, it is natural that conflicts arise. The government represents the oppressor, the blind follower of rules and the one that imposes limits to individual freedom. Governmental authoritarianism symbolises everything postmodern individuals set out to eradicate. The dismissal of authority that is implicit in these protests leads to the dismissal of the laws conceived by that same authority. This belief in a temporary suspension of the law promotes the liberation of aggressive and instinctual drives, creating an almost state of anarchy, which is illustrated by the violence of the riots that occurred in several countries during the pandemic (Wood et al., 2022). The expression of these drives is as strong as the anger experienced towards authority. The anger against “the father” is not new, it is as ancient as the beginning of family (Frankl, 2001); however, the freedom to express this anger is a novelty.

Postmodern culture does not just allow the gratification of desires, but also encourages fighting against more powerful figures. The disinhibition of the Oedipus conflict, expressed in the struggle against authority, was associated by Freud with a lack of religion and a lack of moral restraint. Freud (1923) claimed that religion and morality, i.e. “the higher nature of men” (p. 34) were acquired through the process of mastering the Oedipus complex.

Nonetheless, the liberation of anger towards authority is not enough to conclude that there has been a liberation of the instincts of the id. On the contrary, in the context of this pandemic, the censorship over the id was significant for all those who have complied with COVID restrictions and recommendations. Most of our social needs had to be suppressed or highly controlled; our ritualistic behaviours such as touching our nose and mouth also had to be self-monitored; even our feelings of fear, anxiety, doubt and panic had to be repressed not to become overwhelming. Some claim there has been media censorship during COVID that mainstream television channels and newspapers have opted for not publishing divergent opinions and not interviewing experts who might have had a different opinion than the one being promulgated by the government, regarding COVID-19 rules as well as vaccines (Chang et al., 2022). Additionally, whenever divergent opinions did appear in the news, they were swiftly erased from the web and made impossible to find.

This amount of self-control and censorship over instincts and drives evokes the traditional Freudian super-ego instead of a postmodern super-ego if we consider Frankl’s claims. Nonetheless, if we consider Žižek’s description of the postmodern super-ego, censorship is an integral part of it, even though the censored content has changed drastically in the sense of an imposition of freedom, equality, respect, self-expression and enjoyment. Žižek often gives the example of political correctness as a new form of censorship. Contrary to what is argued by Frankl, it is clear that there is still a strong pressure to conform to certain values and ideals, the question is who is the new authority we serve? Why do we serve it? How does it communicate and what are its strategies and final purpose?

### Reflexivity, Uncertainty and Anxiety

The COVID-19 pandemic shattered the perspective of stability and comfort that hovered over the Western World, awakening the imminence of death, and rallying its countries against a common and fierce enemy. The expression “war against COVID-19” and the use of wartime strategies to fight the virus were broad and extremely frequent (Benzi & Novarese, 2022). This pandemic has triggered war time responses not just from governments and law enforcers but also from the population.

*On Reflections on war and death* (Freud, 1918), Freud’s depiction of the Western world in the outbreak of World War I is strangely consistent with the morale during the COVID-19 pandemic. He describes, on the one hand, a scenario of confusion, lack of reliable information and uncertainty about the future and, on the other hand, an unavoidable confrontation with our own death, the shattering of the immortality illusion. According to Frankl, it was precisely the destruction displayed by the two world wars that triggered the emergence of a new postmodern super-ego, marked by disappointment, apprehension and scepticism towards the future.

In the pandemic scenario, while the confrontation with death could have, on the one hand, awakened a survival instinct and caused an alignment with the government’s strategies and regulations, it also could have, on the other hand, caused a rejection of the fear, a denial of the death threat, feelings of uncertainty and confusion and a repression of reality. This uncertainty was not exclusively due to not knowing when and if the pandemic would end but it was also due to the information that circulated in the news and the formal and informal advice on ways of preventing infection, ways of contracting the virus, etc. This conflicting information did not only originate in the news and in social media but also in scientific articles (Nagler et al., 2020). All new information was to be interpreted and decoded, leading to debates and generally ending in scepticism and in the sole possible conclusion: that there is no way of knowing the truth. The result of this climate of uncertainty was a division in society.

As soon as the restrictions to fight the pandemic started to be implemented, this issue became a political matter more than a health situation. This pandemic divided the Western population into two facets: those in favour of full compliance to governments’ directives, considering them as good, reasonable, necessary, altruistic and a matter of public health and those who rejected them, considering them a violation of civil liberties, a despotic and paternalistic attempt to control the population through the instigation of fear, putting economical and political interests ahead of the people’s interest. This conflict reignited the underlying political bipolarisation of several countries, torn between liberal and conservative political parties. In some countries, like Brazil and the USA, this struggle was more important than in most European countries.

The fact that a public health problem was transformed into a partisan issue, where every person had an opinion and a different interpretation of the facts illustrates Žižek’s concept of reflexivity. Scientific articles were discussed as if everyone had become a medical expert, disinformation was at its peak, conspiracy theories spread fast and truth was nowhere to be found.

More recently, Žižek (2020) addressed another issue brought by the pandemic that is intrinsically connected to the postmodern super-ego and the anxiety it can cause. He describes the issue of boredom, which marked the long periods of lockdown, as a conflicting experience with the commands of the postmodern super-ego, the imperative to enjoy every moment of every day to the fullest, resulting in the types of anxieties referred to in the previous chapter — FOMO and FOBO. Just as the super-ego did not stop emitting orders, it also did not stop censoring thoughts and behaviours.

The responses to COVID-19 do not merely illustrate the postmodern super-ego’s intolerance for dead time, and they also highlight the moral uncertainty that troubles today’s Western societies. Since we no longer live in compliance with tradition and that there is no commonly accepted code to guide our social and moral behaviour, all our impulses, experiences and actions have become matters to be reflected on and debated to exhaustion. Why do we select certain information to focus on, and why do we prefer one analysis to another? After being confronted with multiple opinions, it becomes difficult to take a definitive stand for or against something or someone.

This reality, instead of annihilating the super-ego, as George Frankl would posit, only augments the level of anxiety associated with constant doubting, wishing and ruminating. If an individual is arrested for not complying to coronavirus rules, for protesting for his idealised rights, it only raises questions such as “Does breaking the law mean I have done wrong?”, “Where does justice lie?” and “If the law has lost all its credibility, how to know right from wrong?”. The abundance of these and many other questions is the result of a culture based on relativism and subjectivity. While under the Freudian super-ego anxiety was caused by the certainties of what we must not do, under the postmodern super-ego anxiety comes from the absolute uncertainty about what we should do.

## Conclusion

This paper has aimed to explore the influence of the postmodern era on the super-ego by examining the hypotheses presented by the several authors. To accomplish this purpose, certain behavioural tendencies were analysed, characteristic of the current social context, that were found to implicate and threaten the foundations of the Freudian super-ego. These tendencies include: the aggression towards paternal authorities; the overturn of traditional and conservative institutions; the potential liberation of instincts of the id; an exemption from prohibitions and restrictions; and the absence of a moral compass, accompanied by a state of general confusion. The example of the coronavirus pandemic is used as a potential portrait of the behavioural changes that implicate the super-ego and that were brought by postmodernity.

On the one hand, the behavioural duality found during the pandemic between obedience and disobedience makes it an exceptionally challenging example to be able to infer a potential alteration of the Freudian super-ego. Individuals were under an unusual amount of pressure since their safety and well-being was being threatened. On the other hand, this challenging scenario can also be very informative since it forces the truth to surface by pushing every boundary and dissipating cultural superficialities.

First, this analysis allows the rejection of Frankl’s hypothesis of an annihilation of this structure, particularly, since the ego-ideal still manifests itself and the feelings of guilt, anxiety and shame persist. The persistence of these feelings suggests the super-ego maintains the same pattern through which it exerts control over the ego and the id.

Secondly, it upholds the claim that there has been an overturn of traditional values and parental authority, manifested in the break of the bond with the authority, allowing the expression of aggression towards powerful figures and suggesting a liberation from censorship of at least this specific instinct of the id.

Thirdly, the break of the bond with the paternal authority, by causing a rejection of traditional values, forced individuals to enjoy their freedom and auto-determination, compelling them to form opinions about every matter and elaborate personal rules of conduct. The postmodern super-ego emerges in the struggle to reject the introjection of the parental super-ego and to form a personalised code of do’s and don’ts. Postmodern individuals follow their own ideal representations and manage the satisfaction of their instincts as they see fit, within a difficult and personal balance of transgression and restraint.

Finally, this psychic agency now conjugates harshness with liberalism, and it is moved by the unconditional obligation to be free, happy and true to itself. What remains in the place of the super-ego is the persistence of guilt, anxiety and shame, and a strong will to conform to a set of ideals inspired by non-authoritarian role-models.

This conclusion points to an idea already mentioned in the first chapter, namely, the idea that the ego-ideal has taken the place of the super-ego. Lieberman’s clinical observations support this claim. She concludes that “the new super-ego” is fundamentally more narcissistic and subjective — being a good person corresponds to conforming to one’s ideal of good, wealth and beauty rather than from good and altruistic consensual deeds such as helping others, following the ten commandments, respecting the elderly or being kind. Individuals are now guided by a different set of standards and values that seem to affect the nature and the functioning of the super-ego.

The contribution of this paper is the deconstruction of the functioning of the super-ego and its revision in the light of twenty-first century culture. Particularly, regarding the break of the bond with paternal figures; the empowerment of the ego-ideal and of the feeling of shame; and a reflection on the state of moral uncertainty and subsequent new social anxieties.

As a final point, there are clear limitations to the research presented in this paper. As in all open-ended research, interpretations are limited since personal experience and knowledge may influence observations and conclusions. Thus, even though an effort has been made to cross-reference all hypotheses with quantitative data, conclusions and causality cannot be objectively verified. In addition, there is undoubtedly a lot of room for further research on the super-ego and on how permeable to culture it can be. It is essential to understand how the phenomena of morality, obedience, repression and guilt have evolved since Freud and Klein and how that may change the way psychoanalysis approaches the postmodern individual in terms of research, as well as in the clinical practice.

## Data Availability

Data sharing not applicable to this article as no datasets were generated or analysed during the current study.
